# Mississippi Valley Dental Association

**Published:** 1884-06

**Authors:** F. W. Sage


### MISSISSIPPI VALLEY DENTAL ASSOCIATION.

FORTIETH ANNUAL MEETING, HELD AT CINCINNATI, MARCH 5TH AND 6TH, 1884.

PHONOGRAPHICALLY REPORTED FOR THE INDEPENDENT PRACTITIONER.

BY F. W. SAGE, D. D. S.

(Continued from page 258.)

The next subject opened for discussion was: "What valuable medicinal agents have been introduced into dental practice within the last three years?"

*Dr. Taft*—The peroxide of hydrogen is one of the agents recently employed in dental practice, about which inquiry is made. My actual experience with it is limited. It is a very good disinfectant. Disinfectants act by breaking up the elementary or binary constituents of a decomposing substance, and so render them inert. It is, however, of little value as an antiseptic. A small amount of vitiated matter will oftentimes keep up decomposition, and a disinfectant will restore the affected tissues to healthy action. The offensive matter is the cause of the irritation, and the vital function is often fully restored by the free use of a disinfectant. Peroxide of hydrogen is a disinfectant, but not an antiseptic. Some may attribute the return to healthy action of the affected parts to its antiseptic effects, but that is not always necessary.

*Dr. Smith*—How does peroxide of hydrogen act?

*Dr. Taft*—By the liberation of oxygen, and its combination with the offensive matter in the abscess destroys its offensive character.

Another agent to which I before referred as a remedy for sensitive dentine, is worthy of mention as a remedy in facial neuralgia. That is Menthol. It is distilled from the oil of peppermint. A few crystals placed in a watch-glass, moistened with a drop or two of chloroform and tincture of aconite, and applied to the face, often act beneficially. A convenient way to use it is to make it into an ointment with vaseline, and apply over the track of the aching nerve. I have also used it in my own case in the treatment of lumbago. It affects the sensory nerves immediately. It should be prepared immediately before using, to prevent the chloroform from evaporation.

Salicylic acid is another agent—not very new, it is true—which is useful in devitalized teeth, where there is a low grade of inflammatory action. It is a disinfectant, and an antiseptic also, to some extent. Do not apply it where there has been recent or acute inflammation about a root. In some such cases I have found it aggravated the trouble. Where there is a low, chronic form of irritation, kept up by fluids or gases, I know of nothing better to use than salicylic acid. Iodoform is also a good disinfectant. Its odor is very objectionable. That may be masked, but it is a question whether it can be done without impairing its efficiency. Resorcin is also good, and is less offensive than iodoform. Oil of Eucalyptus is an antiseptic, and to some degree a disinfectant also. It may be used where a stimulus is not required. I do not think it has any advantage over other agents used. I doubt whether it is better than Thymol, which has long been used. Listerine is simply a disinfectant. It may be used for deodorizing about the office, or in the mouth. I have my patients wash their mouths with it sometimes, when their breath is very offensive. It is efficient, and is easily applied. I use a little, a teaspoonful, in a glass of water, for rinsing my own mouth at night. It is better for the uses for which it is designed than Permanganate of Potash. The latter agent is objectionable on account of its staining.

*Dr. H. A. Smith*—The remedies Dr. Taft has mentioned are all valuable. I beg leave, however, to differ with him as to Salicylic acid. It destroys tooth substance. I have found that it acts to disintegrate tooth structure. Dr. Taft stated correctly that it was the oxygen in the Peroxide of Hydrogen which is the active principle. Years ago it was used for bleaching teeth. It is nothing new. A great many medicines are put upon the market as disinfectants. It is not wise to rely implicitly upon any single one of these in all cases. Carbolic acid and glycerine are often very efficient. I give Permanganate of Potash the preference over Listerine for bad cases. Creosote, I think, deserves a high place among the list of dental remedies. It prevents putrefaction, but as a deodorizer we know but little of its virtues. Carbolic acid, we know, will sterilize beef, kill the spores, prevent fermentation, and, with the exception of Bichloride of Mercury, it is the best agent for arresting putrefaction. Dr. James is the only dentist I know who has dared to use this latter agent.

*Dr. James*—It is true that I did venture to use it in one instance [laughter] and the patient still lives. Not only that, but I think the agent accomplished just what was wanted.

*Voices*—How did you use it? What did you use it for?

*Dr. James*—I used it to cure an abscess, in accordance with the suggestion of a physician. One grain to the ounce of water pumped into the root-canal on a broach.

*Dr. Van Antwerp*—That would be about one-fifth of one per cent. Physicians are using it in the proportion of one part in eight hundred parts of water.

*Dr. Smith*—I think that if we can use this safely we have a good thing. Its stimulating property will prevent fermentation or putrefaction. It kills like "sure shot."

*Dr. Wright*—Do not die in the house. [Laughter.]

*Dr. Van Antwerp*—It is interesting to hear that the dentists are giving this remedy their attention. The medical profession are experimenting to kill the bacillus by inhalation.

*Dr. H. A. Smith*—It is claimed, you are aware, that in the use of some filling materials peculiar therapeutic effects are produced, whereby dental caries is more certainly arrested. I recall some recent experiments of Koch, than whom there is no better authority, to determine the value of the salts of mercury for disinfecting, especially the chloride of mercury. Of the eighteen drugs experimented with, corrosive sublimate stands first in value as a true disinfectant. This chloride, he found, not only retards putrefaction, but actually prevents it. An aqueous solution of five per cent. or less, kills the spores and prevents their further development. The statement is frequently made that whenever amalgam is used as a filling material, a chloride of mercury is formed in the mouth, which frequently produces the physiological effects of the metal, and hence the use of amalgam is denounced. On the other hand, it is stoutly denied that any constitutional effects ever result from the use of amalgam. The fact cannot, however, be denied, that amalgam being placed in a tooth, the walls about the filling gradually assume a dark hue. This discoloration is ascribed to a deposition of some salt in the dental tubules. It is believed, as I have stated, that chloride of mercury is so deposited. If this be present, may not the so-called therapeutic action of amalgam fillings be due to the marked antiseptic and disinfecting property of the corrosive sublimate? A solution of this salt is white, whereas the color of the tooth is changed to a blackish hue. But, since the filling leaks, may not the discoloration be attributed to the presence of other salts of the metal? for example, sulphureted compounds. It is true, probably, of nearly all the alloys in use, that they leak, and yet they have very good tooth-preserving qualities. This is indeed a common feature of several of the plastics—an unexplained phenomenon—that they are not water-tight, and still teeth are saved by their use. It has frequently been stated upon this floor that caries never recurs about an oxy-chloride of zinc filling so long as the filling remains intact. And yet Prof. Flagg, who may be regarded as most excellent authority, says the oxy-chloride preparations shrink notably. So also of the gutta-percha preparations. Indeed, when we consider the very attenuated form which water may assume, it seems almost incredible that water may be almost absolutely excluded from the cavity, even by gold filling, especially if the operation be difficult or complicated. How exceedingly thin must be the film of water that floats away in the air in the form of a soap-bubble! A recent calculation by Sorby shows that 3,700,000,000,000,000 molecules of water are contained in one cubic thousandth of an inch! (Three quadrillions seven hundred trillions!) These figures are surely sufficient to alarm those of us who practice conservative dentistry on the principle that teeth are saved by excluding moisture from the cavity of decay.

*Dr. Taft*—I opine that the answer will be found in this explanation: The vapor of mercury is exceedingly volatile; it is almost impossible to make the joints of a retort so tight that it will not pass through. It penetrates the dentine of the tooth frequently, and discolors it. It passes into the tubules and oxidizes, and the particles of oxide are larger than the particles of the vapor, and so the ends of the tubules become closed by them. These particles thus form a shield against the agents producing decay, and the result is their effectual arrestation. It is stated that some amalgams do not thus blacken the dentine. That may depend either upon the fact of the teeth being very dense, or the amalgam may be so prepared that the white vapor of mercury does not escape, and so there is no penetration and subsequent discoloration of the tubules. But, notably, soft teeth and the teeth of young persons are liable to become black when amalgam is used.

*Dr. Smith*—You will usually find chlorine free in the mouth in such cases, and what then prevents the formation of bichloride of mercury? I think there *is* that combination.

*Dr. Taft*—I hardly think so. Oxygen is in far greater abundance, even granting that free chlorine exists, and it asserts its affinities first of all. I do not imagine that bichloride of mercury is ever produced by the presence of amalgam fillings. But if it is, it is one of the strongest arguments against using amalgam fillings that could be advanced. That would prove conclusively that amalgam is a subtle poison, and its use might then well be characterized as criminal. But always, precisely this formation of the oxide of mercury precludes the possibility of any other salt being formed.

Now, again; as to the distinction between a disinfectant and an antiseptic. Oxygen is the chief agent in decomposition, and any agent which will prevent the occurrence of that process is called a disinfectant. A disinfectant acts in one of two ways: it either takes away one of the elementary constituents of the offensive matter, or it separates those constituents and leaves them free to form new compounds of an inoffensive nature. What is decomposition? You destroy the vitality of a tissue, and oxygen presently asserts its affinities, being no longer held in abeyance by the vital force, and the tendency is to dissolution of the tissues. Since oxygen is a necessary factor in the case, if you exclude it from the surface exposed to its influence, you prevent decomposition. If, for example, you apply carbolic acid, it coagulates the albumen of the tissue, forming an eschar, so that oxygen has no access to the tissue, and no decomposition occurs. That is a part of the antiseptic process. Whether it be by a solid or a liquid covering, the result is the prevention of decomposition, provided the germs be effectually excluded.

*Dr. How*—My idea of the distinction between a disinfectant and an antiseptic is like this: a disinfectant is a policeman who steps in and arrests one of the offending agents after the disturbance has fairly commenced, and so breaks up a row. [Laughter.] The antiseptic is another policeman, who takes possession and preserves the peace on the first sign of an impending disturbance. One is corrective, the other preventive.

*Dr. Smith*—Admitting, as has been asserted, that oxygen gets to work more promptly than do other liberated gases in the mouth, it is beyond controversy that other gases seek their affinities just as positively and unerringly, if less conspicuously. Now, why is it presumed that chlorine is an exception in this instance? Those who accept the theory of the formation of hydrochloric acid in the mouth as an agent of decay, do not hesitate to speak of the presence of free chlorine in the oral fluids. Why will not this unite with mercury if it be present? I am not yet convinced that that does not occur. It is well known that English amalgam blackens dentine in a very marked manner. Sullivan's amalgam does this. Now, an amalgam which blackens *reasonably well* is better than one which does not blacken at all.

*Dr. Taft*—There occurs to me an insurmountable obstacle to Dr. Smith's suggestion—that formation of mercury cannot be sufficiently volatile to pass into the tubules. The only way, indeed, that I can see how this blackening is effected, is by the infiltration of the vapor of mercury. Chlorine has a stronger affinity for the dentine than for mercury.

*Dr. Hoff*—Peroxide of Hydrogen has proven very effective in my experience of six months' use of it. It has taken the place of creosote. I like it because of the facility with which it can be applied. Used in a syringe it does not, like other agents, cauterize the soft tissues. I used it in one instance in treating two molars about which the process had been lost by necrosis. Could not get through the roots. Filled with oxy-chloride of zinc, and treated the parts externally by injections with the syringe, once repeated. The cure is apparently complete.

At the conclusion of the discussion, the Committee on Obituaries reported a resolution on the death of Dr. T. L. Buckingham, as follows:

This Association desires to place upon record its sense of the great loss which the profession has sustained by the loss of Prof. Thos. L. Buckingham. We may not hope by any form of words to express the value of his faithful and life-long devotion to the educational and practical interests of the whole profession, or of his most estimable character as a man, but we offer this heartfelt tribute in token of our sincere appreciation of what he has wrought for us. We wish also to convey to his bereaved family our sympathies with them in this time of their sore affliction and irreparable loss. The Secretary is instructed to transmit to the family a copy of these resolutions in behalf of the Mississippi Valley Association of Dental Surgeons, in annual association at Cincinnati, March 6th, 1884.

W. S. How,
G. W. Smith,
J. Taft,
*Committee.*

The annual election of officers of the Association for the ensuing year resulted as follows:

| | |
|---|---|
| President—Dr. WILLIAM VAN ANTWERP, . . | Mt. Sterling, Ky. |
| Vice-President—Dr. F. W. SAGE, . . . . . | Cincinnati, O. |
| 2d Vice-President—Dr. A. L. EMMINGER, . . | Columbus, O. |
| Recording Secretary—Dr. A. G. ROSE, . . . . | Cincinnati, O. |
| Corresponding Secretary—Dr. A. BERRY, . . | Cincinnati, O. |
| Treasurer—Dr. FRANK HUNTER, . . . . | Cincinnati, O. |

